# Extracts from the Records of the Boston Society for Medical Improvement

**Published:** 1857-05

**Authors:** F. E. Oliver

**Affiliations:** Secretary


*Extracts from the Records of the Boston Society for Medical Improvement.* By F. E. Oliver, M. D., Secretary.

Feb. 23d.—*Growth of a Horn.*—Dr. H. G. Bigelow showed a specimen of horn removed at the Hospital, by Dr. Cabot. The patient, aged 42, married, had always had a large mole where the tumor was situated, at the top of the back, half an inch to the right of spinal column. She had never had an extraordinary feeling in it until about a year ago. It then began to be sore, when she lay on her back, or when her dress bore upon it. The bunch then commenced to grow, until it had risen a little above the level of the surrounding tissues. It is hard, about the size of a filbert, and immovable on account of its firm, deep attachments. She had recently had acute lancinating pains in and about the growth. Her general health had been good, nor was there any cancerous disease in the family.

The tumor was enclosed above in a sort of sac, which enveloped its horny tip. This surmounted a mass of concave epithelial layers, arranged like a pile of cups, corresponding to the matrix of a nail. The length of the tissue constituting the matrix was three-fourths of an inch; that of the horn, one-fourth an inch; the whole being of the diameter of a swan's quill and buried in the fat. The horn was about to perforate the cutis by ulceration.

Feb. 9th.—*General Affection produced by the application of Atropine to the Eye.*—Dr. Bethune mentioned the case.

On finding that the pupil did not dilate on application of a solution of atropine containing one grain to the drachm, an application of double this strength was made three times successively. In the afternoon the patient was attacked with delirium, there being also uncertainty in his gait, with absence of sleep and difficulty of swallowing. On the day but one after, he had another attack of delirium, somewhat resembling *delirium tremens*, seeing imaginary persons in the room. On the following day he was well.

Feb. 23d.—*Unusual Sequela of Scarlet Fever.*—Dr. Cabot mentioned the case.

The patient was the father of two children who had both had this disease. The eldest of the two had last taken it, and during the first week of its progress the father was seized with the sore throat. There was no eruption; there was, however considerable prostration, fever, and pain in the bones. The urine was cloudy, and dark colored, but contained no albumen at the time Dr. C. saw him. In various parts of the body small spots soon after appeared, which became painful, and were followed by suppuration, having the characteristics of that following phlegmonous inflammation. There were two of these spots on one arm, and one on the other; also one over the region of the sacrum as large as the palm of the hand. The patient is still under treatment.

Feb. 23d.—*Recurrent Tumor of the Orbit*—Dr. Bigelow showed a portion of the diseased growth removed by him from the patient whose case was reported at the first meeting in January, and who had been operated upon by himself before that date. The upper surface of the bony orbit itself being affected, Dr. B. did not attempt another operation for the removal of the disease. Although, as Dr. B. remarked, the disease is microscopically innocent, it is clinically malignant, and nothing further can be done for the patient.

Feb. 23d.—*Ovariotomy, and the operations for Ovarian Disease.*—A question of Dr. Jackson as the probable cause of the ill success that had attended the recent operations for ovarian disease in this city, led to some discussion in regard to the relative propriety and success of the various operations which have been and are still in vogue in this disease.

Dr. Cabot alluded to the success that had attended the operation in Paris, by injection of the tincture of iodine, and the favor it had consequently found with French surgeons. Little trouble seemed to follow the operation, except, in a few cases, some degree of inflammation, and, in some instances, the iodic intoxication. In view of the great array of successful cases, he was inclined to consider this mode of operating as on the whole the safest and best. He spoke of the quantity of the tincture of iodine used by the French surgeons, as important in preventing the putrid suppuration so liable to follow this operation.

Dr. H. B. Bigelow had formerly operated twice without success, and for a number of years has declined to do the operation of ovariotomy. It put the patient in great danger, and, from the frequency of the operation out of town, it is probable that there are unrecorded cases which would increase its estimated mortality. In this class of cases, the surgeon encounters a large number of patients, young and old, solicitous to be relieved from a disease which subjects them to discomfort, and sometimes to annoying suspicions; many being still in the enjoyment of tolerable health, and with a fair prospect of life for some years. In fact, these last are the most favorable cases for ovariotomy. There is no surgical operation at once so frequently offering itself, and so largely and immediately fatal, to patients in a comparatively comfortable state. The injection of iodine and the permanent canula are both useful chiefly in the case of unilocular cysts, which are quite rare compared with the usual multilocular form of disease, and not to be distinguished with certainty from the latter. Dr. B. referred to a fatal case resulting from each of these methods of treatment. Tincture of iodine is composed of iodine and alcohol. Alcohol alone would probably be as efficacious an injection in a variety of cases usually treated by injection of the tincture of iodine. The frequent and careful washing out of a large ovarian cyst with a bland fluid after operation, to remove decomposing or acrid discharge, or even of the peritoneal cavity, as recently detailed in a case of Prof. Peaslee, doubtless tends to procrastinate or avert a fatal result.

Dr. Warren remarked that these cases in London had generally been in the hands of specialists, and that he had not seen the operation performed in any of the principal hospitals of that city. He had himself operated by the injection of iodine, but without effect. He alluded to a case in which Dr. Simpson, of Edinburgh, operated in this way. Eight ounces of the tincture of iodine were thrown in. The operation was followed by pain, swelling, and other constitutional symptoms of considerable severity, from which, however, the patient recovered. He thought it important to ascertain, if possible, the result in these cases after the expiration of a considerable time.

Dr. Coale alluded to the fact that the time required for the sac to re-fill after tapping, varies in different cases. In one case in which he operated, in May, 1853, no further operation had as yet been required. Nine pints of a dark-colored and tenacious fluid were thrown off.

Dr. Gay said that some cases recover from simple tapping, and others from local and constitutional treatment. He mentioned cases on record where recovery followed the rupture of the cyst; and referred to certain cases reported some time since by Dr. Channing. With regard to the treatment by the injection of the tincture of iodine, he remarked that this is now undergoing a trial in England; that while in some cases it had been attended with success, in others no benefit followed. In a few cases, the peculiar effects of the iodine had been observed, but these had been recovered from in a few days. In reference to the usual mode of tapping, he said that where this is done, death is pretty sure to follow, sooner or later, in most cases. He had tried tapping and allowed the canula to remain, recently, in two cases (see Boston Med. and Surg. Journal, Vol. LV., p. 409,) in neither of which were there any very unpleasant symptoms; in one, none whatever. To the latter operation there seem to be two important objections. 1st, The impossibility of ascertaining beforehand, with certainty, whether the cyst be single or compound, the former being alone likely to be benefited by the operation; there being also the same objection to the operation; by the injection of iodine. 2d, The long and tedious suppurative discharge which the patient is necessarily obliged to undergo. He alluded to several cases of treatment by the canula, which had proved successful in the hands of Dr. Trowbridge. He further stated that he should not hesitate to employ the iodine treatment in cases of a single cyst.

Dr. Gay was evidently opposed to the operation by extirpation, except under favorable circumstances, and said that he had recently performed this operation only at the urgent request of patients.

March 9th.—*Disease of the Brain; Convulsions; Death; Autopsy.*—Case reported by Dr. C. D. Homans, who also showed the specimen. Mr. ——, aged 46 years, had always enjoyed good health, though the child of parents both of whom died of consumption. At 16 years of age, he fell and fractured the bones of his nose, the result of which was an inability to breath through that organ afterward. He was a merchant in Calcutta until within a few years, but of late had resided in Boston. During his residence in the East Indies, he had an attack of what was called "sun-stroke," which confined him to the house for some time.

On March 4th, 1856, while sitting at his desk, writing, he had a severe convulsion, from which he recovered in the course of a few hours so as apparently to be as well as ever. He pursued his business, and continued well till March 31st, when he had two fits while at his club, more severe than that of the 4th inst. His struggles were violent, requiring the efforts of several persons to control them. The next dayhe was out as usual.

April 6th, at the house of a friend he had two convulsions, and was carried home; immediately after which, his mental faculties became disordered; memory of names of persons and things impaire l; he imagined himself away from home, in another house, where he could stay but a limited period, &c. For some days he refused to eat, and the urine and fæces were discharged involuntarily. He soon, however, took food again regularly, and at the end of about three weeks he had recovered strength sufficiently to walk down stairs, having kept his bed previously. He then appeared insane, and it was necessary always to have a male attendant with him. April 25th he escaped from the house, and bought a country seat at auction, the purchase of real estate being one of the principal subjects his mind seemed to dwell upon. He would sometimes absolutely refuse nourishment, and again soon after take it; the tongue was slightly coated; pulse somewhat accelerated; there was a tendency to ulceration in the mucous membrane of the mouth; the skin was natural save that there was some heat about the head. There was at times long continued constipation.

June 15th.—At this time he appeared much better; he took his meals regularly, and his mind seemed to be tending toward a normal state. He was ordered to abstain entirely from animal food, but otherwise to live as those about him did.

July 4th, he seemed worse; complained of pain in his head, walked unsteadily, reeling backward; mind as bad as ever. Toward the last of this month he began to amend, and continued slowly to do so through August, so that by the middle of September he appeared to have perfectly recovered.

February 17th, 1857.—Mr. ——, the patient, came home with a violent pain in the head, accompanied with drowsiness. He arose the next day as well as usual, and continued so until February 21st, when on awakening he complained of great pain in the head; this pain he always referred to the vertex. He kept his room, and at 5, P. M., had two severe convulsions; at 8, another; and at 5, A. M., Sunday, a fourth. During this day he was well enough to come down stairs, and in the evening sat in his room conversing with an intimate friend. His mind was perfectly clear; he gave directions about his affairs, in case of death, which he anticipated might ensue at any time. At 9½, P. M., he lost his consciousness, and did not regain it.

February 23d, at 2½, A. M., the convulsions recurred, and continued with short intermissions until Friday, at 9, P. M., numbering 170 in this time, according to the report of the watcher. Sometimes they would run into each other, and at other times there would be an interval of five, ten or fifteen minutes. A few times there were longer intervals, after which the attacks were more severe. From 12, Thursday noon, to midnight, there were 70 convulsions. They were general, affecting however, the right arm and leg decidedly more than the left. The left eye was very much injected. After 9, P. M., Friday, the patient had no more convulsions, but lay in a comatose state till Sunday, March 1st, at 6, P. M., when death took place. On Friday morning there was a lucid interval, just long enough for him to recognize and shake hands with his family.

The treatment consisted in the administration of sulphuric ether to control the convulsive attacks; and nourishments and stimulants were employed whenever there was an opportunity for them to be swallowed.

*Autopsy*—Tuesday, March 3d, 41 hours *post mortem.*

The *body* was of medium size, and not at all emaciated; *rigor mortis* well marked. In the *thorax* and *abdomen*, nothing abnormal was noticed.

*Head.*—Nothing remarkable was found in the *brain*, save at the anterior part of the *left hemisphere*, just below where the skull is generally sawed in removing the calvaria, an inch and a half above the upper edge of the orbit; here the brain over the surface of one and a half to two inches in diameter, was so clearly adherent to the dura mater that the pia mater and a thin layer of the cerebral substance were left behind in the removal of the organ. The *dura mater* itself was readily raised from the bone, and no evidence of injury or disease was noticed in the latter.

In the *cortical substance*, which had adhered to the membranes, and extending a short distance into the white matter, were too distinct, round, firm, yellowish-white, somewhat granular-looking masses, the largest half an inch in diameter. They adhered closely to the surrounding substance, which for several lines in every direction was filled with minute yellow points. Portions of the white substance, half an inch below the principal mass, had a peculiar translucent appearance, as if formed of delicate parallel fibres, in the interstices of which serum had been infiltrated.

*Microscopic Examination* by Dr. C. Ellis. In the yellow masses, nothing was seen but amorphous granular matter, and minute molecules or globules. In the portions around, which contained the yellow points, were many fat globules, while the normal tissue, if it existed, was entirely obscured. In the translucent portions were extremely delicate fibres, some of them parallel, some of them forming an irregular reticulated structure, minute fat globules every where abounding.—*Boston Med. and Surg. Joarnal.*

